# Correlation between intrafractional motion and dosimetric changes for prostate IMRT: Comparison of different adaptive strategies

**DOI:** 10.1002/acm2.12359

**Published:** 2018-06-03

**Authors:** Nami Saito, Daniela Schmitt, Mark Bangert

**Affiliations:** ^1^ Department of Medical Physics in Radiation Oncology German Cancer Research Center Heidelberg Germany; ^2^ National Center for Radiation Research in Oncology (NCRO) Heidelberg Germany; ^3^ Heidelberg Institute for Radiation Oncology (HIRO) Heidelberg Germany; ^4^ Department of Radiation Oncology Heidelberg University Hospital Heidelberg Germany

**Keywords:** adaptive treatment, electromagnetic tracking, IMRT, intrafractional motion, prostate cancer

## Abstract

**Purpose:**

To retrospectively analyze and estimate the dosimetric benefit of online and offline motion mitigation strategies for prostate IMRT.

**Methods:**

Intrafractional motion data of 21 prostate patients receiving intensity‐modulated radiotherapy was acquired with an electromagnetic tracking system. Target trajectories of 734 fractions were analyzed per delivered multileaf‐collimator segment in five motion metrics: three‐dimensional displacement, distance from beam axis (DistToBeam), and three orthogonal components. Time‐resolved dose calculations have been performed by shifting the target according to the sampled motion for the following scenarios: without adaptation, online‐repositioning with a minimum threshold of 3 mm, and an offline approach using a modified field order applying horizontal before vertical beams. Change of D95 (targets) or V65 (organs at risk) relative to the static case, that is, ΔD95 or ΔV65, was extracted per fraction in percent. Correlation coefficients (CC) between the motion metrics and the dose metrics were extracted. Mean of patient‐wise CC was used to evaluate the correlation of motion metric and dosimetric changes. Mean and standard deviation of the patient‐wise correlation slopes (in %/mm) were extracted.

**Results:**

For ΔD95 of the prostate, mean DistToBeam per fraction showed the highest correlation for all scenarios with a relative change of −0.6 ± 0.7%/mm without adaptation and −0.4 ± 0.5%/mm for the repositioning and field order strategies. For ΔV65 of the bladder and the rectum, superior–inferior and posterior–anterior motion components per fraction showed the highest correlation, respectively. The slope of bladder (rectum) was 14.6 ± 5.8 (15.1 ± 6.9) %/mm without adaptation, 14.0 ± 4.9 (14.5 ± 7.4) %/mm for repositioning with 3 mm, and 10.6 ± 2.5 (8.1 ± 4.6) %/mm for the field order approach.

**Conclusions:**

The correlation slope is a valuable concept to estimate dosimetric deviations from static plan quality directly based on the observed motion. For the prostate, both mitigation strategies showed comparable benefit. For organs at risk, the field order approach showed less sensitive response regarding motion and reduced interpatient variation.

## 1 INTRODUCTION

Intrafractional motion induces potential miss‐dosage in targets and overdosage in adjacent normal tissues. For prostate treatments with intensity‐modulated radiation therapy (IMRT), intrafractional motion has been mitigated with optimized margins^1^, ^2^, ^3^ or endorectal balloons.^4^ In the last decades, online motion detection including MV portal imaging,^5^, ^6^ cone beam computed tomography (CT),^7^ ultrasound,^8^, ^9^ electromagnetic transponders,^10^, ^11^, ^12^ and magnetic resonance imaging^13^, ^14^ have been introduced.

On the one side, the resulting motion information facilitated the development of real‐time mitigation techniques using a couch,^15^, ^16^, ^17^ multileaf‐collimator (MLC)‐tracking,^18^, ^19^ or gated delivery.^20^, ^21^ On the other side, it is possible to reconstruct the delivered dose, that is, a four‐dimensional (4D) dose distribution, based on the actually observed motion during the treatment.^22^, ^23^, ^24^ Although motion of the prostate during radiotherapy has been detected^25^, ^26^ and adaptive strategies have been applied for prostate patients,^2^, ^15^, ^27^ a comprehensive comparison of motion management strategies is still pending.^28^

Here, we present a coherent analysis of the correlation between intra‐fractional prostate motion and dosimetric changes induced by the motion considering both target coverage and organ at risk (OAR) sparing metrics. Real‐time motion data^29^, ^30^ stemming from 21 patients were used, and different motion mitigation approaches were compared. Our study provides valuable guidance to implement clinical decision support regarding the adequate motion mitigation strategy for prostate IMRT patients. Furthermore, we introduce two innovations: (a) a novel motion metric which quantifies the target motion relative to the actual delivered beam (DistToBeam) and (b) the strategy of changing the field order (AngHV) that can be applied for many clinical scenarios in IMRT.

## 2 MATERIALS AND METHODS

### 2.A Patient data

Data for 21 prostate patients who received step‐and‐shoot IMRT with a Siemens Artiste treatment device equipped with a 160‐leaf MLC (Siemens AG, Munich, Germany) in 35 fractions at our institution was acquired. Informed consent was obtained from all included individual participants. The study was approved by the ethics committee of the medical faculty at our university. The prostate was defined as an integrated boost volume and the treatment plan was optimized with the goal of a prescribed median dose of 76 Gy with an enclosing isodose of at least 95% (72.2 Gy). The prostate was expanded by a margin of 7 mm including the base of the seminal vesicles to construct the planning target volume (PTV) with a prescribed dose of 70 Gy with an enclosing isodose of at least 95% (66.5 Gy). Treatment planning was performed with our in‐house clinical treatment planning systems VOXELPLAN and KonRad which facilitate a singular value‐decomposed pencil beam algorithm.^31^ The immanent inaccuracies of pencil beam algorithms compared with more sophisticated approaches in heterogeneous media were assumed negligible for prostate treatments.^32^ Nine coplanar equiangular fields with a 6‐MV photon beam with a flattening filter were used (Gantry angles at 200°, 240°, 280°, 320°, 0°, 40°, 80°, 120°, 160° in a clockwise order according to the IEC 61217 standard). The patient was positioned in head‐first supine position using a head rest, a knee rest, and a foot rest. There was no dietary protocol, but the patients were asked for having an empty rectum and a full bladder during the planning CT and treatment fractions. For each patient, the clinically applied treatment plan was used as a reference to simulate different motion mitigation strategies. The average number of MLC segments per plan was 81, and the total number of fractions was 734 in our cohort.

### 2.B Motion metrics

**Figure 1:**
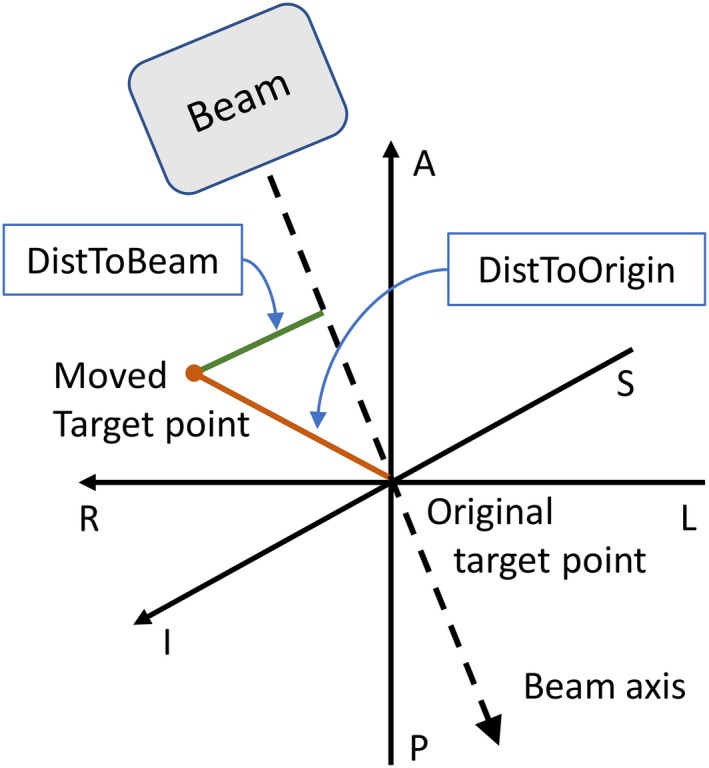
Schematic drawing of the beam geometry for one segment with an original and a moved target point. The three axes are indicated and the corresponding distances DistToOrigin and DistToBeam. The patient‐related axes correspond to a patient in head‐first supine position.

Target motion was monitored with the Calypso 4D localization system (Varian Medical Systems Inc., Palo Alto, CA)^33^, ^34^ during the treatment by detecting the center of mass of 3 electromagnetic transponders implanted in the prostate of each patient. Time‐correlated data of 3D trajectories of the target and the beam delivery record were available for 717 fractions. The average duration of the trajectory recording was 10 min per fraction. For each fraction, the displacement of the target was sampled once per MLC segment by averaging the trajectory data during the irradiation of the respective MLC segment. In total, 59 385 motion samples were obtained considering five different motion metrics describing the displacement of the prostate: the 3D distance from the original target point (DistToOrigin); the displacement components in left–right (LR), posterior–anterior (PA), and superior–inferior (SI) direction related to a patient in head‐first supine position; and the distance from the beam axis (DistToBeam) in beam's eye view; see Fig. 1.

To extract intrafractional motion for this study, the initial displacement has been set to zero with an offset correction based on average motion data considering 30 s before the onset of treatment for each fraction. This approach corresponds to perfect patient positioning. Missing data (0.4% in the recorded data) were imputed by linear interpolation if the gap was during the irradiation and lasted less than a few seconds.

### 2.C Dose quality metric

4D dose distributions that represent the dose accumulation on the moving anatomy were calculated for each fraction by first shifting the target point location relative to the patient CT for all beam segments and subsequently summing the resulting dose distributions for all segments. Therefore, we use an in‐house developed 4D dose reconstruction software based on the same dose calculation algorithm as the original treatment planning procedure. The dose reconstruction was validated in measurements with a moving phantom.^30^ In order to perform the dose calculation for an entire 35 fraction treatment course, missing fractions were substituted by randomly choosing other fractions of the same patient.

The reference dose quality in the form of the PTV D95, prostate D95, bladder V65, and rectum V65 was extracted from the reference plan for each patient (average values; 64.8 Gy, 70.3 Gy, 17.4 % and 23.2 %, respectively). As an additional check of our workflow, we recalculated the original plans with the 4D‐dose calculation by specifying all target motion to be zero. Note that the average coverage of the PTV and the prostate does not meet the optimization goals laid out in Section 2.A. Due to conflicting objectives regarding OAR sparing, the goals regarding target coverage had to be relaxed for individual patients. Dosimetric quality changes induced by the motion were analyzed based on the relative change of D95 (V65), that is, ΔD95 (ΔV65) in percent per fraction and percent per treatment course.

### 2.D For 4D dose reconstruction, we consider three different motion scenarios

#### 2.D.1 Free motion scenario

The actual treatment was delivered without intrafractional motion adaption (NoCorr). Based on the time correlated beam delivery information, the observed target motion was sampled per delivered beam segment and used for the reconstruction of the dose distribution that was actually delivered during the treatment.

#### 2.D.2 Repositioning scenario

Real‐time adaptation scenarios with a couch‐shift technique have been simulated with repositioning thresholds of 3, 5, and 7 mm (Rep3, Rep5, and Rep7, respectively) by using the sampled target motion of the patients. In this scenario, the repositioning has been applied if the sampled displacement exceeds the threshold at any orthogonal direction. Therefore, the displacement of all components is immediately set to zero simulating infinitely fast patient repositioning. After repositioning, the sampled motion continues relative to the applied centering. Since the duration of repositioning in this simulation is neglected, the beam delivery timing and ending of the sampled motion coincides with the NoCorr case.

#### 2.D.3 Field order modification scenario

For this offline motion management scenario, we simulate a treatment that starts with horizontal fields and finishes with vertical fields (AngHV), that is, we apply a gantry angle order of 280°, 80°, 120°, 240°, 320°, 40°, 160°, 200°, and 0°, instead of the clockwise order. This approach is motivated by the following considerations: (a) the mean displacement of the target is larger along PA and SI than along LR direction,^25^, ^26^ (b) the displacement increases with treatment time,^29^, ^35^ (c) the vertical direction of the beam would not introduce significant change in the dose delivery, if the target is moved along PA because in beam's eye view, the field is still covering the target geometry. Horizontal fields, in contrast, are more sensitive to the dominant target motion in PA and SI directions. The AngHV approach was compared with an original clockwise order case (AngCW) using the same average time sequence from our study cohort (2 s beam on; 2 s beam off for MLC change, 20 s beam off for gantry angle change). This ensured the same conditions for AngHV and AngCW to detect purely the angle order effect. Again, missing motion data were imputed from existing records of the same patients. Note that AngHV and AngCW define a new time sequence; consequently, motion data had to be imputed for different patients (4 of 717 fractions) than before.

### 2.E Correlation analysis

The Pearson product–moment correlation coefficient (CC) between the dose metric (ΔD95 or ΔV65) and the motion metric (LR, PA, and SI, DistToOrigin, DistToBeam) was computed for every patient individually. The number of data points underlying the CC calculation was up to 35 (number of fractions). Subsequently, the mean values of all 21 patients were extracted. The mean value of the CC in the NoCorr scenario was used to identify the motion metric with the best correlation to the dosimetric endpoints.

Linear regression was performed to quantify the sensitivity of all dose metrics with a slope (i.e., dose metric per motion metric) in %/mm. A motion management strategy with low motion sensitivity would result in less dose changes from the static case for a given motion (i.e., a smaller slope). Consequently, the strategy can be considered more robust against motion. The standard deviation of the slope in patient statistics was included as error estimate.

## 3 RESULTS

### 3.A Motion metric

Mean, standard deviation (SD), minimum and maximum of the sampled target displacements are listed in Table 1. The maximum displacement in the motion metric DistToOrigin, DistToBeam, LR, PA, and SI was 25.4, 17.7, 10.4, 19.9, and 15.2 mm, and the mean displacement ±SD was 1.7 ± 1.5, 1.4 ± 1.3, 0.1 ± 0.7, −0.6 ± 1.4, and 0.6 ± 1.4 mm, respectively, considering all 59385 samples for the NoCorr case. With real‐time repositioning, the displacement was reduced for the smaller thresholds. For the simulated delivery of AngCW and AngHV, the mean displacement and SD resulted in the same magnitude in DistToOrigin, LR, PA, and SI due to the offline nature of AngHV that does not adapt the target position; only DistToBeam showed differences.

**Table 1:** Statistics of the different motion metrics. The target displacement was sampled for all 59 385 segments in 734 fractions of 21 patients

Scenario	Statistics of the displacement (mm) in motion metric				
	DistToOrigin	DistToBeam	LR	PA	SI
NoCorr					
Mean	1.7	1.4	0.1	−0.6	0.6
SD	1.5	1.3	0.7	1.4	1.4
Minimum	0.0	0.0	−5.0	−12.0	−15.2
Maximum	25.4	17.7	10.4	19.9	10.4
Rep3					
Mean	1.3	1.0	0.0	−0.5	0.4
SD	0.8	0.8	0.6	0.9	0.9
Minimum	0.0	0.0	−3.0	−3.0	−3.0
Maximum	4.8	4.6	3.0	3.0	3.0
Rep5					
Mean	1.6	1.3	0.1	−0.6	0.5
SD	1.2	1.0	0.7	1.2	1.2
Minimum	0.0	0.0	−4.0	−5.0	−5.0
Maximum	6.9	6.9	4.2	5.0	5.0
Rep7					
Mean	1.7	1.4	0.1	−0.6	0.6
SD	1.4	1.2	0.7	1.3	1.3
Minimum	0.0	0.0	−5.0	−7.0	−6.7
Maximum	9.6	8.8	4.2	6.9	7.0
AngCW					
Mean	1.6	1.3	0.1	**−**0.5	0.5
SD	1.4	1.2	0.6	1.3	1.3
Minimum	0.0	0.0	**−**7.2	**−**9.6	**−**15.3
Maximum	19.5	15.6	4.0	11.8	7.8
AngHV					
Mean	1.6	1.2	0.1	**−**0.5	0.5
SD	1.4	1.1	0.6	1.3	1.3
Minimum	0.0	0.0	**−**7.2	**−**9.6	**−**15.3
Maximum	19.5	15.4	4.1	12.1	7.8

### 3.B Dose quality metric

The changes in the dose metrics calculated for all scenarios are listed in Table 2 for both the fractional dose and the cumulative dose per patient. For the fractional dose in NoCorr, the change of dose metric ΔD95 for PTV and prostate was −2.0 ± 3.5% and −0.9 ± 2.1 % ranging from −24.9 to 2.2% and −26.7 to 1.8%, respectively. The negative sign indicates less dose in NoCorr than for the static case. For both the PTV and the prostate, the minimum values showed large deviation from the static case. For Rep7, 5, and 3, the dose deviation decreases as the threshold decreases. ΔD95 of AngHV shows improvement of the minimum value compared with AngCW from −16.9% to −10.1% for the prostate, while it stays comparable for the PTV with about −24%. For the cumulative dose, the PTV ΔD95 and the prostate ΔD95 were within 5% for all patients in all scenarios.

**Table 2:** Statistics of dose metrics (i.e., ΔD95 or ΔV65) for fractional and cumulative dose as change of D95 or V65 relative to static cases

	Scenario	Change (%) in dose metric			
		PTV ΔD95	Prostate ΔD95	Bladder ΔV65	Rectum ΔV65
		Mean ± SD (min–max)	Mean ± SD (min–max)	Mean ± SD (min–max)	Mean ± SD (min–max)
Fractional dose (n = 734 fractions)	NoCorr	−2.0 ± 3.5 (−24.9–2.2)	−0.9 ± 2.1 (−26.7–1.8)	9.4 ± 19.7 (−85.3–189.3)	−7.1 ± 27.2 (−93.3–480.8)
	Rep3	−0.9 ± 1.3 (−6.6–3.1)	−0.4 ± 0.9 (−6.9–2.5)	6.3 ± 9.8 (−30.9–54.1)	−6.2 ± 13.2 (−81.2–76.8)
	Rep5	−1.5 ± 2.0 (−11.9–2.2)	−0.7 ± 1.5 (−12.5–1.8)	8.6 ± 15.0 (−51.4–102.8)	−7.3 ± 18.4 (−94.7–157.5)
	Rep7	−1.9 ± 2.9 (−24.3–2.2)	−0.8 ± 1.7 (−16.9–1.8)	9.5 ± 17.5 (−53.4–111.3)	−7.7 ± 21.2 (−89.1–219.4)
	AngCW	−1.7 ± 2.9 (−23.0–2.4)	−0.8 ± 1.7 (−16.9–2.2)	8.0 ± 16.7 (−86.6–122.4)	− 6.0 ± 25.8 (−94.5–485.7)
	AngHV	−1.0 ± 2.3 (−24.5–3.6)	−0.5 ± 1.2 (−10.1–3.3)	5.4 ± 12.3 (−51.0–85.9)	−1.5 ± 18.5 (−81.1–233.8)
Cumulative dose (n = 21 patients)	NoCorr	−1.2 ± 1.5 (−4.2–1.4)	−0.3 ± 1.1 (−4.4–0.7)	9.1 ± 10.4 (−6.4–45.5)	−8.1 ± 10.6 (−42.0–15.3)
	Rep3	−0.4 ± 0.9 (−2.2–1.5)	0.0 ± 0.5 (−1.7–0.9)	6.1 ± 6.0 (−4.7~25.7)	−6.6 ± 9.0 (−38.5–12.6)
	Rep5	−0.9 ± 1.2 (−2.8–1.4)	−0.2 ± 0.9 (−3.5–0.8)	8.4 ± 9.2 (−5.4–41.1)	−8.0 ± 10.9 (−47.5–13.7)
	Rep7	−1.1 ± 1.4 (−3.8–1.4)	−0.3 ± 1.0 (−3.9–0.7)	9.2 ± 10.1 (−6.2–43.9)	−8.5 ± 10.9 (−44.8–14.8)
	AngCW	−1.0 ± 1.3 (−3.7–1.3)	−0.2 ± 0.8 (−2.9–0.8)	7.6 ± 8.0 (−5.9–33.2)	− 6.8 ± 8.4 (−30.5–14.3)
	AngHV	−0.4 ± 1.0 (−2.7–1.2)	0.1 ± 0.5 (−0.6–1.2)	4.6 ± 4.5 (−5.0–14.1)	−2.7 ± 5.1 (−8.6–10.2)

Bladder V65 increased for all motion and correction scenarios in comparison to the static case, while the rectum V65 showed an overall decrease. Both is valid for fractional and cumulative doses; see Table 2. This may be explained by a general drift of the entire anatomy toward the posterior direction. For the fractional doses, ΔV65 was 9.4 ± 19.7 % for bladder and −7.1 ± 27.2 % for rectum in NoCorr. Repositioning scenarios show a reduction of the SD of ΔV65 and minor changes in the corresponding mean values. For the cumulative dose, the bladder ΔV65 of 9.1 ± 10.4 % of NoCorr case was improved by the reposition approach of Rep3 to 6.1 ± 6.0 %. The bladder ΔV65 was improved to 4.6 ± 4.5 in AngHV compared to of 7.6 ± 8.0 % in AngCW. For the rectum ΔV65 in the cumulative dose, Rep3 showed −6.6 ± 9.0 % instead of −8.1 ± 10.6 % of NoCorr. AngHV resulted in a bladder ΔV65 of −2.7 ± 5.1 % compared with −6.8 ± 8.4 % in AngCW.

### 3.C Correlation coefficients

Mean values of the patient‐wise CCs are listed in Table 3. The PTV ΔD95 and the prostate ΔD95 showed the strongest correlation with DistToBeam and comparable CC values with DistToOrigin. The repositioning approaches showed less correlation for smaller thresholds. In all scenarios, ΔV65 of the bladder has a moderate correlation in PA direction and a strong correlation in SI direction. The rectum ΔV65 has the highest correlation in PA direction and a moderate correlation in SI direction. No correlation has been detected with LR metric.

**Table 3:** Mean Pearson product–moment correlation coefficient (CC) between dose metric and motion metric in 21 patients

Dose metric	Scenario	CC for motion metric				
		DistToOrigin	DistToBeam	LR	PA	SI
PTV ΔD95	NoCorrr	−0.73	−0.74	−0.05	0.45	−0.46
	Rep3	−0.53	−0.53	0.00	0.54	−0.53
	Rep5	−0.68	−0.69	0.00	0.57	−0.56
	Rep7	−0.73	−0.74	0.00	0.57	−0.57
	AngCW	−0.71	−0.71	−0.03	0.40	−0.40
	AngHV	−0.64	−0.66	0.02	0.17	−0.22
Prostate ΔD95	NoCorr	−0.63	−0.64	−0.05	0.25	−0.33
	Rep3	−0.30	−0.33	−0.03	0.25	−0.34
	Rep5	−0.49	−0.50	−0.01	0.31	−0.38
	Rep7	−0.62	−0.63	−0.01	0.37	−0.44
	AngCW	−0.60	−0.61	−0.03	0.20	−0.27
	AngHV	−0.51	−0.53	0.08	0.19	−0.24
Bladder ΔV65	NoCorr	0.31	0.33	0.02	−0.84	0.96
	Rep3	0.38	0.40	−0.01	−0.72	0.92
	Rep5	0.42	0.43	0.01	−0.78	0.94
	Rep7	0.44	0.46	0.02	−0.83	0.95
	AngCW	0.29	0.31	0.04	−0.84	0.95
	AngHV	0.28	0.30	0.01	−0.79	0.94
Rectum ΔV65	NoCorr	−0.19	−0.20	−0.06	0.92	−0.80
	Rep3	−0.34	−0.33	−0.04	0.84	−0.69
	Rep5	−0.31	−0.30	−0.06	0.89	−0.73
	Rep7	−0.34	−0.34	−0.06	0.91	−0.79
	AngCW	−0.19	−0.20	−0.05	0.92	−0.80
	AngHV	−0.15	−0.15	0.00	0.72	−0.66

### 3.D Regression slopes

**Figure 2:**
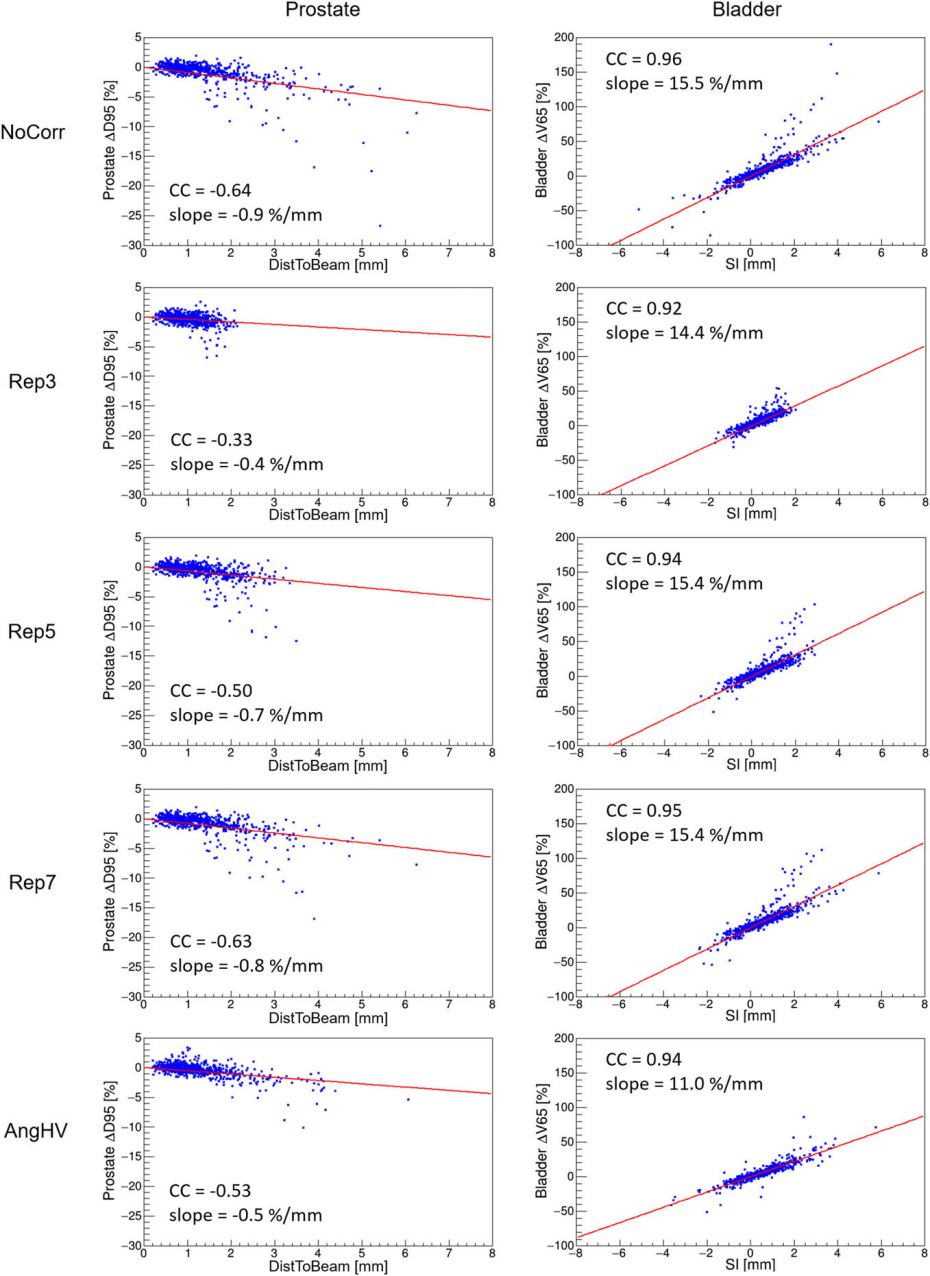
Correlation plots between a dose metric and a motion metric for NoCorr (top), repositioning cases (2nd, 3rd, and 4th rows), and AngHV scenario (bottom). The dose metrics correspond to ΔD95 for the prostate (left) and ΔV65 for the bladder (right). The motion metric corresponds to the mean of DistToBeam for the prostate and SI for the bladder per fraction. The linear regression curves are shown with solid lines; the corresponding correlation coefficients (CC) and slopes are given for each subplot.

Figure 2 shows examples of the correlation plots of the total data set for the selected metric combinations based on the given criteria above. As shown in the figure, the linear regression slopes of these plots are −0.9, −0.4, −0.7, −0.8, and −0.5 %/mm for the prostate and DistToBeam displacement and 15.5, 14.4, 15.4, 15.4, and 11.0 %/mm for bladder and SI displacement in NoCorr, Rep3, Rep5, Rep7, and AngHV, respectively.

**Figure 3:**
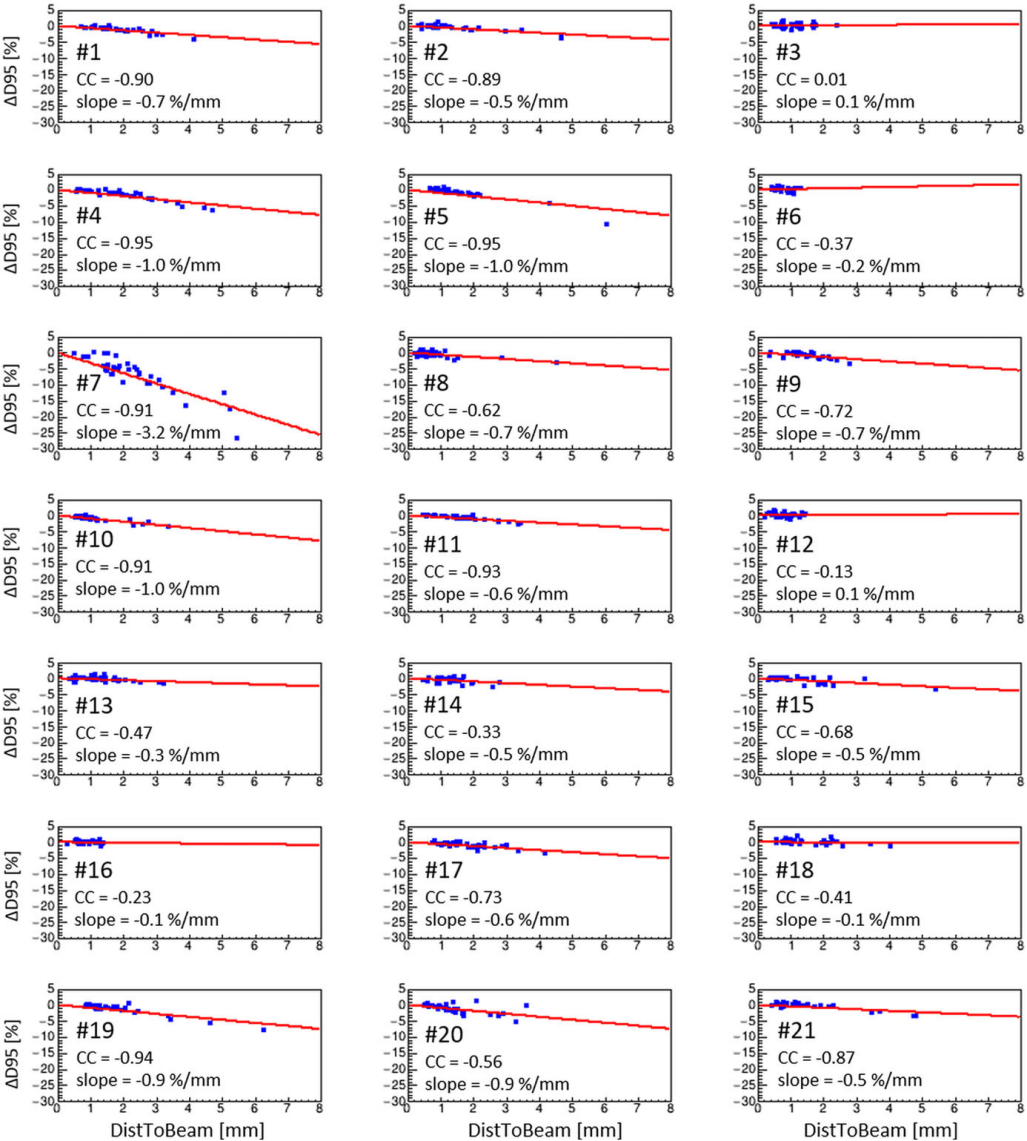
Correlation plot between the prostate ΔD95 and the motion metric DistToBeam, plotted for each patient. The linear regression curves of patient‐wise analysis is shown with a solid line; the corresponding correlation coefficients (CC) and slopes are given for each subplot.

Figure 3 shows an example of patient‐wise correlation plots between the prostate ΔD95 and DistToBeam in NoCorr scenario. As illustrated in the figure, the linear regression of the patient‐wise data shows a more clear correlation than for the total data set averaged over all patients (compare Fig. 1).

The patient‐wise analysis of regression slope is shown in Table 4 for all dose metrics in combination with the motion metric that showed the highest correlation. Both free motion scenarios, NoCorr and AngCW, showed comparable results due to the similarity of the delivery method (i.e., clockwise deliveries without adaptation). Consequently, the slope values of repositioning and AngHV scenarios can be easily compared to a common reference.

**Table 4:** Linear regression slope of the correlation; dose metric per motion metric in %/mm in 21 patient statistics

Scenario	Slope (%/mm)			
	PTV ΔD95 and DistToBeam	Prostate ΔD95 and DistToBeam	Bladder ΔV65 and SI	Rectum ΔV65 and PA
NoCorr				
Mean	−1.6	−0.6	14.6	15.1
SD	1.1	0.7	5.8	6.9
Minimum	−3.5	−3.2	9.5	6.5
Maximum	0.7	0.2	37.2	38.4
Rep3				
Mean	−0.9	−0.4	14.0	14.5
SD	0.8	0.5	4.9	7.4
Minimum	−2.2	−2.0	7.7	6.5
Maximum	0.9	0.3	31.3	42.7
Rep5				
Mean	−1.2	−0.5	14.4	14.9
SD	0.8	0.6	5.8	7.1
Minimum	−2.6	−2.9	9.3	6.5
Maximum	0.6	0.3	36.4	39.3
Rep7				
Mean	−1.5	−0.6	14.6	14.8
SD	1.0	0.6	5.5	6.2
Minimum	−3.0	−2.9	9.5	6.5
Maximum	0.8	0.2	35.1	34.5
AngCW				
Mean	−1.5	−0.6	14.5	15.4
SD	1.0	0.6	5.3	8.2
Minimum	−2.9	−2.8	9.6	7.0
Maximum	0.6	0.2	34.3	44.9
AngHV				
Mean	−1.0	−0.4	10.6	8.1
SD	0.9	0.5	2.5	4.6
Minimum	−2.8	−1.4	7.5	−0.9
Maximum	0.5	0.8	17.1	20.4

### 3.E PTV

For the PTV ΔD95, the most correlated motion metric in the patient‐wise analysis was DistToBeam, with a mean slope (±SD) of −1.6 ± 1.1 %/mm in NoCorr. For the repositioning approaches, the mean values of slope decrease as the threshold decrease with a relatively constant SD (e.g., −0.9 ± 0.8 %/mm for Rep3). The AngHV resulted in the slope of −1.0 ± 0.9%/mm showing a similar reduction in motion sensitivity as Rep3.

### 3.F Prostate

For the prostate ΔD95, the selected motion metric was DistToBeam with mean slope (±SD) of −0.6 ± 0.7 %/mm in NoCorr. With the repositioning approaches, the reduction in the mean CC values is more pronounced compared with the corresponding PTV cases. The slope values of repositioning scenarios show minor reduction (e.g., −0.4 ± 0.5 %/mm for Rep3) from NoCorr case. The AngHV also showed a slope of −0.4 ± 0.5%/mm.

### 3.G Bladder

The CC of the bladder ΔV65 showed the strongest correlation with the SI component with a positive sign. It implies that the target displacement toward inferior (superior) introduces a very linear increase (reduction) of ΔV65 in this study. For this selected combination, mean and SD of the slope in all repositioning scenarios have no significant change compared with the NoCorr case. On the other hand, the AngHV showed reduction in both mean and SD.

### 3.H Rectum

For the rectum ΔV65, the motion metric PA showed the highest correlation among all motion metrics. The target displacement toward anterior (posterior) increases (decreases) the rectum ΔV65 in this study. As it was found in the bladder, the slope values of the rectum did not exhibit a significant change for all scenarios except for AngHV that showed a shallower slope of 8.1 ± 4.6 %/mm.

As it was demonstrated in Figs. 1 and 2 for the prostate, the patient‐specific curves of OARs also introduce variation in the cohort data. AngHV showed smaller variation as measured by the SD of the slope compared with the other scenarios. At the same time, the mean value of the slopes was found to be smaller indicating a reduction in motion sensitivity by AngHV for V65 in both the bladder and the rectum.

## 4 DISCUSSION

Our patient‐wise analysis revealed a clear linear correlation between dose and motion metrics. The motion metric of DistToBeam showed the highest correlation in the correlation analysis for both PTV and prostate for all considered motion mitigation strategies rather than DistToOrigin, as target motion along the beam axis does not affect the target dose significantly. For OARs, the strongest correlation was found in SI and PA direction for bladder and rectum, respectively.

In agreement with a previous study,^24^ the motion metric of mean displacement showed a stronger correlation for the PTV than for the prostate, as it has to be expected. While Langen et al. found a poor correlation with correlation coefficient of −0.26 between mean displacement and CTV coverage, we observed stronger correlation (−0.63) for the corresponding metric, DistToOrigin. This observation equally applies to prostate coverage. The differences can be explained by different margin designs with an integrated boost concept in our study, which directly leads to changes in the prostate coverage in case of target motion due to the directly surrounding dose fall off. Moreover, we established the correlation model using an additional motion metric, that is, DistToBeam, which accounts for the beam geometry in combination with a patient‐wise specific analysis to extract clear correlations.

The scenarios without adaptation, NoCorr and AngCW, showed similar motion sensitivity as measured by the regression slopes, even though the sampled motion amplitudes and timings were different.

The real‐time adaptation scenarios with repositioning, Rep3, Rep5, and Rep7, showed a gradual change in the sensitivity as Rep3 is the most motion insensitive among them. In this study, the Rep7 showed minor improvement in dose, see Table 2, and the motion sensitivity in mean and SD was similar to the free motion cases.

The offline strategy, AngHV, reduced the motion sensitivity to the level of Rep3 for both PTV and prostate. As shown in Table 2, the dose quality for the AngHV was comparable to the Rep3 or Rep5 for PTV and prostate. The AngHV strategy improved dose and reduced motion sensitivity without any additional equipment or treatment interruption. Furthermore, it avoids dose degradation due to motion below the threshold inherent in the repositioning scenario. For OARs, AngHV showed reduction in sensitivity in both mean and SD; on the other hand, the repositioning scenarios did not show significant change on the sensitivity. The beam re‐arrangement will cost time, which seems to contraindicate the aims of this approach based on the assumption of larger motion with longer treatment times. Our dose recalculations were made without considering this elongation because of the absence of this information. This decision can be justified by the following arguments: Assuming a gantry speed of 360°/min, the re‐arrangement will lead to an additional fraction time of approximately 2.5 min to our 10 min for the clockwise order. Based on our own data analysis using 25 prostate patients including the 21 patients here, this may lead to an increase in mean longitudinal and vertical displacement of approximately 0.3 mm. This assumption is based on an extrapolation of the mean displacement of −0.1 mm per minute as observed in the first 9 min within our patient cohort. As the prostate motion tends to be saturated after 9 min,^35^ this may be considered a conservative estimate.

The motion in SI direction was not mitigated in the AngHV approach. That needs to be investigated in further studies possibly applying noncoplanar optimized beams.^36^

The extracted correlations are based on the assumption of rigid patient motion. We understand the possibility of shape changes during the treatment; however, the tracked motion data in this study does not provide information in this regard. Consequently, the estimation of the dosimetric impact was performed under the assumption of a stable anatomy. For the OARs, where we report dosimetric quality indicators that are related to the high‐dose volume, the assumption of a rigid anatomy is particularly relevant for the “prostate side” which is irradiated at higher dose. Potential shape changes far away from the prostate (e.g., due to varying organ filling) have a smaller effect on the reported metrics. More detailed morphological sampling of organ motion, shape change, and combination of the strategies can be investigated in further studies.

In this study, the dosimetric changes on rectum were most beneficial in the free motion scenario (Table 2). The motion adaptation strategies reduced deviations from the planned dose which was higher than in the free motion case (NoCorr). This means, on average, all investigated adaptation techniques increased the dose in the rectum compared with the free motion scenario. This can be explained by the prostate drift toward the posterior direction in the free motion case which moves the rectum into a lower dose region, too.

This study was restricted to step and shoot (SNS) IMRT. The applicability of the field order modification is not straightforward for some modern irradiation techniques (e.g., VMAT, tomotherapy) where irradiation follows specific irradiation trajectories on (helical) arcs. However, in the context of noncoplanar arc therapy,^37^ similar considerations as exercised here could inform the design of optimized irradiation trajectories.

The correlation and motion sensitivity analysis presented in this study offers the possibility to estimate dose on moving organs before the onset of treatment. This may support decision making regarding an adequate motion management strategy before the treatment or allow for a simple estimate of the actually delivered dose after the treatment. While our finding may not easily generalize for alternative margin concepts, the presented methodology is applicable for various margin recipes, adaptation strategies, fractionation schemes, and treatment sites. Our results regarding a modified field order may inform a general discussion of the quality of beam angles in the context of anatomical motion.

## 5 CONCLUSIONS

Correlations between intra‐fractional motion and dosimetric quality have been obtained based on measured intrafractional motion of prostate patients considering treatments with and without motion management. The prostate D95 of the cumulative dose was found to be within 5% from the static treatment plan for all 21 patients in all treatment scenarios. Both online and offline mitigation strategies showed comparable benefit in motion sensitivity regarding the individual fraction doses. For the OARs, the offline approach with field order modification resulted in reduced sensitivity to motion and showed less patient variations in the individual fraction doses.

## CONFLICT OF INTEREST

No conflicts of interest.
